# Impact of Individual Viral Gene Segments from Influenza A/H5N8 Virus on the Protective Efficacy of Inactivated Subtype-Specific Influenza Vaccine

**DOI:** 10.3390/pathogens10030368

**Published:** 2021-03-19

**Authors:** Yassmin Moatasim, Ahmed Kandeil, Ahmed Mostafa, Omnia Kutkat, Mohamed El Sayes, Ahmed N. El Taweel, Maha AlKhazindar, Elsayed T. AbdElSalam, Rabeh El-Shesheny, Ghazi Kayali, Mohamed A. Ali

**Affiliations:** 1 Center of Scientific Excellence for Influenza Virus, National Research Centre, Environmental Research Division, Giza 12622, Egypt; Yasmin.Moatasim@human-link.org (Y.M.); Ahmed.Kandeil@human-link.org (A.K.); ahmed_nrc2000@hotmail.com (A.M.); Omnia.Abdelaziz@human-link.org (O.K.); mohameddiaaelsayes@outlook.com (M.E.S.); Ahmed.Nageh@human-link.org (A.N.E.T.); ra_eny@yahoo.com (R.E.-S.); 2 Department of Botany and Microbiology, Faculty of Science, Cairo University, Gamaa Street, Giza 12613, Egypt; malkhazi@aucegypt.edu (M.A.); tsayed1969@hotmail.com (E.T.A.); 3 St. Jude Children’s Research Hospital, 262 Danny Thomas Place, Memphis, TN 38105, USA; 4 Human Link, Dubai, United Arab Emirates; 5 Department of Epidemiology, Human Genetics, and Environmental Sciences, University of Texas, Houston, TX 77030, USA

**Keywords:** PR8-based influenza vaccine, innate immunity, humoral immunity, vaccine efficacy, H5N8

## Abstract

Since its emergence in 2014, the highly pathogenic avian influenza H5N8 virus has continuously and rapidly spread worldwide in the poultry sector resulting in huge economic losses. A typical inactivated H5N8 vaccine is prepared using the six internal genes from A/PR8/1934 (H1N1) and the two major antigenic proteins (HA and NA) from the circulating H5N8 strain with the HA modified to a low pathogenic form (PR8_HA/NA-H5N8_). The contribution of the other internal proteins from H5N8, either individually or in combination, to the overall protective efficacy of PR8-based H5N8 vaccine has not been investigated. Using reverse genetics, a set of PR8-based vaccines expressing the individual proteins from an H5N8 strain were rescued and compared to the parent PR8 and low pathogenic H5N8 strains and the commonly used PR8_HA/NA-H5N8_. Except for the PR8-based vaccine strains expressing the HA of H5N8, none of the rescued combinations could efficiently elicit virus-neutralizing antibodies. Compared to PR8, the non-HA viral proteins provided some protection to infected chickens six days post infection. We assume that this late protection was related to cell-based immunity rather than antibody-mediated immunity. This may explain the slight advantage of using full low pathogenic H5N8 instead of PR8_HA/NA-H5N8_ to improve protection by both the innate and the humoral arms of the immune system.

## 1. Introduction

Influenza A viruses are differentiated into high pathogenic (HP) or low pathogenic (LP) viruses [1]. H5 highly pathogenic avian influenza (HPAI) viruses were first detected in 1996, and a year later the first human infection case was recorded in Hong Kong as a result of the reassortment of H5N1 with H9N2 low pathogenic avian influenza (LPAI) virus [2,3]. Since then, HPAI H5 viruses became widespread and evolved into several clades. After the 2003 outbreak, clade 2 emerged and further expanded and started to form new H5Nx reassortments. The unified classification of the hemagglutinin (HA) of H5Nx viruses has been designated as clade 2.3.4.4 by the WHO/FAO/OIE H5 Evolution Working Group [4,5].

Due to the segmented nature of the *Orthomyxoviridae* family and the emergence and continuous circulation of HPAI H5 viruses along with other factors, other reassortment events took place in segments other than the neuraminidase (NA) [6].

H5N8 HPAI viruses of clade 2.3.4.4 were first detected in 2014 and further classified into subgroups A and B. While group A is no longer detected, group B has emerged since 2016 and continues to circulate and acquire new reassortant forms mainly driven by wild migratory fowls [7,8,9]. The emergence of HPAI viruses poses a threat to human health and causes massive losses for the poultry industry as it is lethal to chickens. This explains the urgent need to produce a universal flu vaccine that can induce sufficient immune response and long-lasting protection against multiple types of influenza viruses.

The traditional form of most influenza vaccines available commercially are inactivated vaccines. They were formed by attenuating the HA of the HPAI parent virus by removing the multi-basic amino acids from the cleavage site of the HA segment, then using the new modified HA along with the NA of the parent virus and the six internal segments of a low pathogenic virus (such as PR/8 H1N1 virus) to generate a new low pathogenic virus as a vaccine candidate by reverse genetics [1]. In a step toward universal flu vaccine, researchers tried combining segments from many viruses to stimulate a multipotent immune response against multiple influenza viruses.

While using NA and the six internal segments from one influenza virus and the HA from another influenza virus can confer cross protection against both viruses in live attenuated vaccines, using NA from another avian influenza subtype in addition to HA in reassortant inactivated form or without HA provides low cross reactivity and no protection against HPAI challenge infection [10,11].

Influenza virus internal proteins encoding segments were also used by researchers to provide cross protection. PB1, M1, and NP viral proteins produced higher protection in mice when compared to HA alone [12,13]. This could be due to T cell response stimulation [14]. Immunization with M2 also did not protect chicken from death after HPAI virus challenge infection [11].

The role of each segment of the influenza virus vaccine strain in the induction of protection from lethality in avian species is not yet clear. In a previous study [15], we noticed that H5 commercial vaccines had a role in partial protection despite being antigenically different which might be due to the internal genes and not to HA. In this study, we aim to determine the contribution of each of the segments of H5N8 virus [16] isolated from wild birds to reduce lethality due to challenge infection with the parental HP strain after vaccination with each of the eight segments reassorted with seven remaining segments of the PR8 H1N1 virus.

## 2. Results

### 2.1. Rescue of Reassortant Viruses

**Figure 1:**
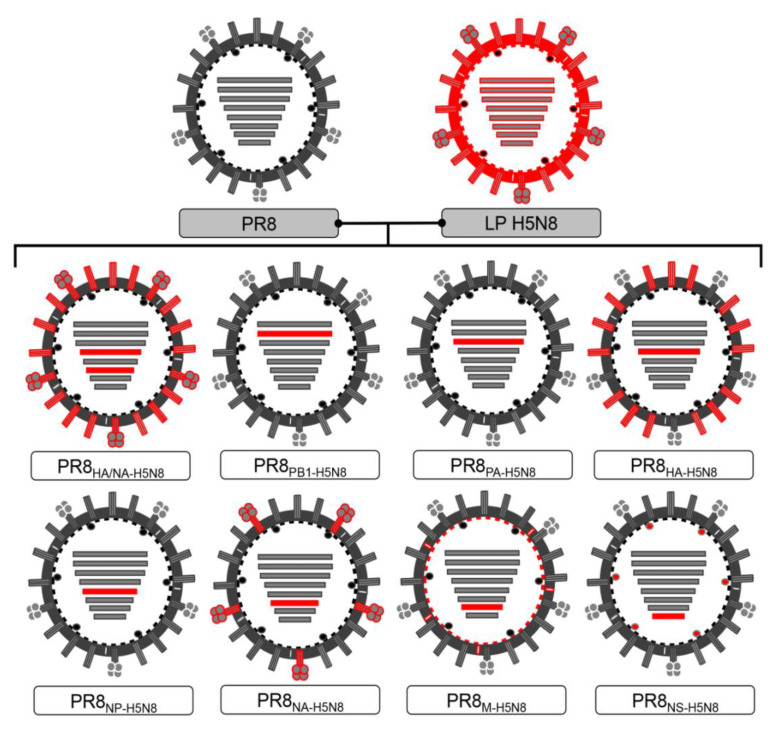
List of successfully generated reassortant viruses using reverse genetics. Plasmids of A/Puerto Rico/8/34 (H1N1, PR8) are shown in grey while A/green-winged teal/Egypt/871/2016 (H5N8) plasmids are shown in red.

The eight gene segments of the HPAI A/green-winged teal/Egypt/871/2016 (H5N8) virus were successfully amplified and cloned in pHW2000. H5N8 virus’s cleavage site multi-basic amino acid sequence (PLREKRRKR/GLF) was altered into a monobasic form (ETR/GLF) as previously described [17]. In order to investigate the role of each segment of H5N8, a set of PR8-based vaccines expressing the individual segments from an H5N8 strain were rescued and compared to the parent PR8 and LP H5N8 strains and the commonly used PR8_HA/NA-H5N8_ (Figure 1). Despite that the PB2 of H5N8 was successfully cloned, confirmed by sequencing, and that the LP H5N8 virus was successfully rescued by reverse genetics, the vaccine form of (PB2 H5N8 + 7PR8) was not rescued after several trials which could be explained by genetic incompatibility. Therefore, this vaccine candidate form was excluded.

Rescued reassortant viruses and parental H1N1 PR8 and HPAI H5N8 were propagated for two passages and titrated by hemagglutination (HA) assay. Viruses were individually adjusted using phosphate buffered saline to 6 log_2_ HA/50 µL.

### 2.2. Immunogenicity and Protection Capacity of Each Form of Inactivated Vaccines

Four-week-old chickens were tested for the presence of maternal protection against PR8 H1N1 and H5N8 parental viruses using hemagglutination inhibition (HI) assay. All chickens were negative for maternal antibodies at the start of vaccination.

#### 2.2.1. Humoral Immunity

**Figure 2:**
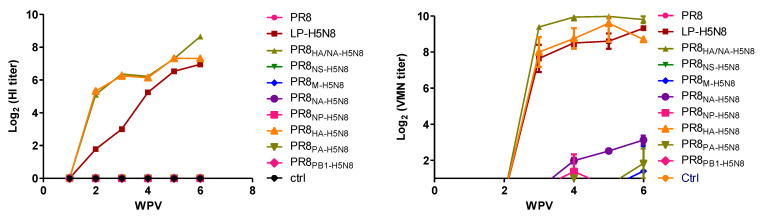
Virus Microneutralization (VMN) and hemagglutination inhibition (HI) assays for evaluating antibody responses at different weeks post vaccination (WPV) to AI H5N8 virus in vaccinated chicken groups with full LP-H5N8, PR8HA/NA-H5N8, and seven groups including one segment of H5N8 virus plus seven segments of PR8, and the control group.

Post vaccination, serum samples collected each week were subjected to HI and viral microneutralization (VMN) assays against the HPAI H5N8 parental virus. Chickens started to develop an antibody response at 2 weeks post vaccination (wpv) only in the three groups containing HA of H5N8 (LP-H5N8, PR8_HA-H5N8_, and PR8_HA/NA-H5N8)_. The antibody titers tested by HI in the collected serum increased with time to 6 wpv in the three groups of vaccinated chickens containing HA of H5N8. All other vaccines based on the six remaining segments showed very low (PR8_NA-H5N8_) to no titers in VMN to 6 wpv (Figure 2).

#### 2.2.2. Survival Rate and Virus Shedding

**Figure 3:**
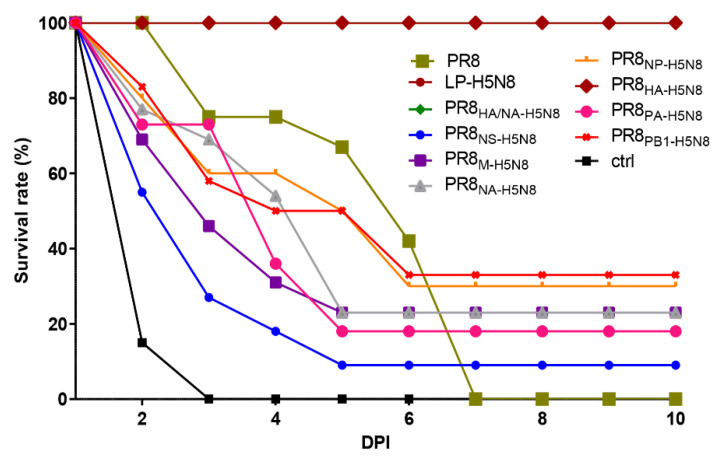
Survival rate of vaccinated chicken groups different days post infection (DPI) with the HP H5N8 virus.

Chickens were then challenged with the parental HPAI H5N8 virus, and virus shedding was titrated in oral and cloacal swabs using EID_50_. Viral RNA was detected in the lung using real time RT-PCR. LP-H5N8, PR8_HA-H5N8_, and PR8_HA/NA-H5N8_ vaccinated groups had a zero mortality rate (Figure 3), and the viral shedding in both oral and cloacal samples was less than 0.5 log_10_ EID_50_. PR8 vaccinated group showed zero survival rate similar to the non-vaccinated control chickens, and viral shedding was higher than 2 log_10_ EID_50_. The chickens vaccinated with the PR8 vaccine showed extended 4-day survival post infection compared with the control non-vaccinated challenged group. The six other H5N8 segments-based vaccinated groups showed a range of survival rates (9–33%). The highest survivals were in groups vaccinated with PR8_NP-H5N8_ and PR8_PB1-H5N8_ (30 to 33% survival), followed by the PR8_PA-H5N8,_ PR8_M-H5N8,_ PR8_NA-H5N8_ (18 to 23%), and PR8_NS-H5N8_ groups (9%) (Figure 3).

**Figure 4:**
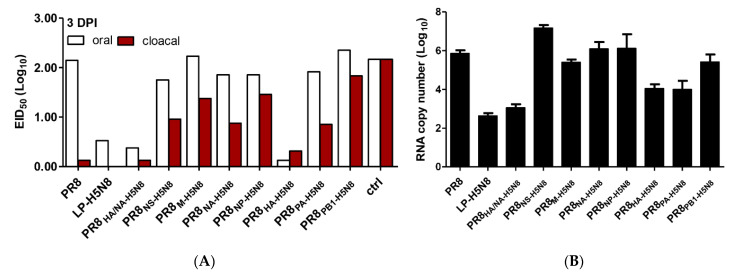
Viral shedding in vaccinated chicken groups at day 3 post challenge with the HP H5N8 virus in oral and cloacal swabs (**A**) and lungs (**B**).

Viral shedding in challenged vaccinated groups (PR8_HA-H5N8_, PR8_HA/NA-H5N8,_ and LP-H5N8) was significantly lower than other groups in both oral and cloacal swabs at 3 days post infection (dpi) (Figure 4A). PR8_HA-H5N8_, PR8_PA-H5N8_, PR8_HA/NA-H5N8,_ and LP-H5N8 vaccinated groups showed the significantly lowest levels of viral shedding in the lungs of challenged chickens as shown in Figure 4B.

### 2.3. Cytokine Analysis

**Figure 5:**
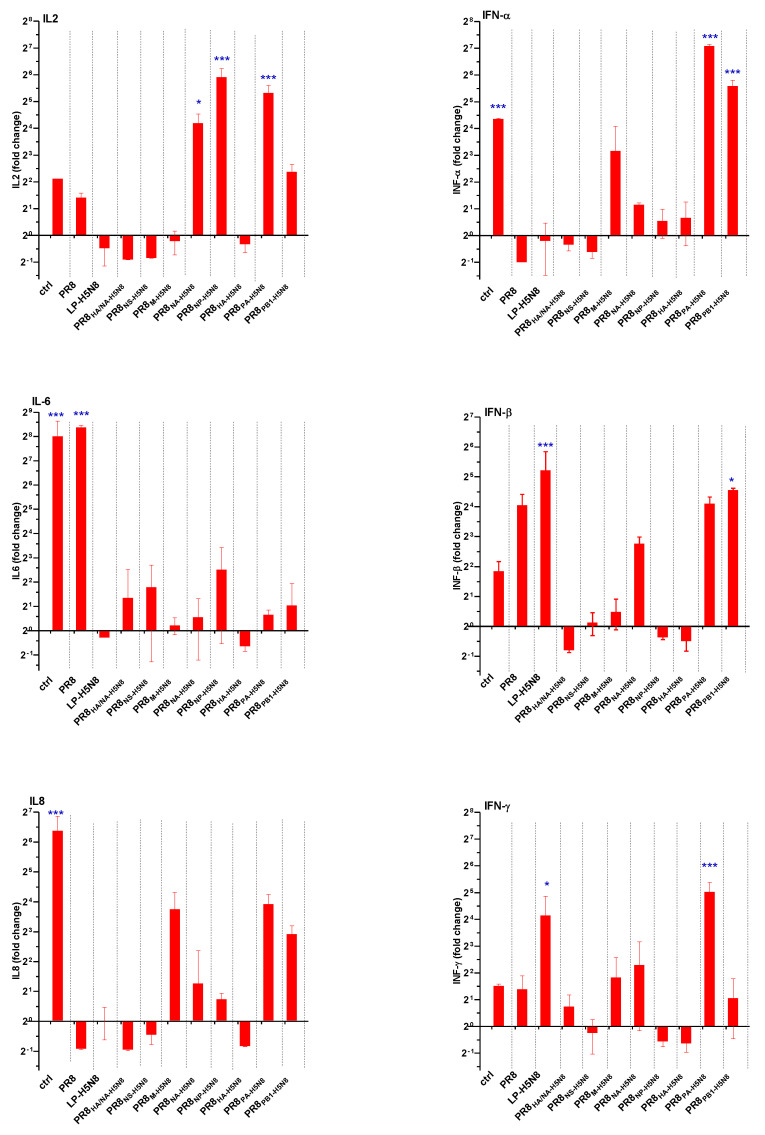
Cytokine expression levels in the lungs of vaccinated chicken groups post challenge with the HP H5N8 virus normalized to vaccinated non-challenged chickens. The control group is unvaccinated–challenged chickens normalized to unvaccinated–uninfected chickens. Stars represent the statistical significance in expression levels of marked groups (* indicates *p* value < 0.05; *** indicates *p* value < 0.001).

Lungs were collected from three chickens at 6 wpv and 3 dpi, and then RNA expression levels of tested cytokines were detected using RT-PCR. Here, we measured the expression levels of type one immune response cytokines responsible for enhancing cellular immunity, such as the interleukin 8 chemokine (IL-8), proinflammatory local and systemic response cytokines (IL-6), type 1 interferons (IFN-α and IFN-β) responsible for antiviral activity, type 2 interferon (IFN-γ) produced by T cytotoxic lymphocytes [18], and interleukin 2 (IL-2) produced by T helper 1 cells, involved predominantly in cellular immune response (Figure 5).

In Figure 5, the control bar represents the expression fold change between the infected–unvaccinated group and the uninfected–unvaccinated groups. All other bars represent the difference in expression between the infected–vaccinated groups and the uninfected–vaccinated groups.

A significantly higher expression of IL-2 was observed for the PR8_PA-H5N8,_ PR8_NP-H5N8,_ and PR8_NA-H5N8_ groups. A significantly higher expression of IL-6 was observed in the control and the PR8 groups, compared to all groups vaccinated with vaccines harboring one or more H5N8 segments. IL-8 was significantly elevated in the control group only. The control, PR8_PA-H5N8,_ and PR8_PB1-H5N8_ had elevated IFN-α while the LP-H5N8 and PR8_PA-H5N8_ had elevated IFN-γ. IFN-β showed significantly elevated expression in PR8_PB1-H5N8_ and LP-H5N8 groups, compared to all other groups.

## 3. Discussion

This study aimed to determine the role of each segment in vaccination in inducing protection when challenged with the parental virus in poultry. Previous studies showed that vaccines including the HA glycoprotein generate protection and neutralizing antibodies against the homologous virus, and immunization with inactivated vaccines having HA alone are capable of inducing a strong immunity and complete protection following challenge infection [11]. Similarly, the vaccine including the HA segment was protective in our experiments. In this study, the vaccination with LP-H5N8 showed the ability to induce expression of both cellular and humoral immune responses in challenged chickens, unlike the vaccination with only HA (PR8_HA-H5N8_) or HA and NA (PR8_HA/NA-H5N8_)_._

Similar to previous studies [10,11], in our experiments the H5N8 NA vaccination in a whole virus vaccine did not induce strong protection (less than 30%). The vaccination using six other internal H5N8 influenza virus segments induced partial protection (ranging from 9% in PR8_NS-H5N8_ to 33% in PR8_PB1-H5N8_) despite having low detectable levels of neutralizing antibodies. This protection might be due to the stimulation of type one immune response and thus strong cellular immunity rather than stimulating type 2.

In general, immune response type I is mainly correlated with stimulating strong cellular immunity and also with the production of T-cytotoxic and T-helper 1 lymphocytes, along with the up regulation of IL-2, TNF-α, and IFN-γ. Immune response type II is more correlated with (but not restricted to) inducing T helper 2 lymphocyte production to produce IL-4, leading to the stimulation of a humoral immune response, downregulation of IFN-γ, and the production of IL-2 cytokine to induce antibody synthesis and proliferation of natural killers. In this study, PR8_PB1-H5N8_, PR8_PA-H5N8,_ and PR8_M -H5N8_ showed upregulation of cellular antiviral response (IFN type 1 and 2 cytokines) and induction of natural killers and T helper 1 cell proliferation (IL-2) (except for PR8_M -H5N8_) when challenged, even more than vaccines harboring an HA segment. These three groups also showed elevated levels of IL-8 chemokine (or CXCL8), causing increased recruitment of neutrophils and ensuring the maintenance of the inflammatory reaction [19].

All infected groups showed reduced expression of the major inflammatory cytokines (IL-6) compared to the control infected group and the PR8 vaccinated group. Under certain conditions, IL-6 is produced by infected dendritic cells (DC) and macrophages to stimulate the acute phase response proteins that have direct antiviral activity and to activate T and B cells. Vaccination with LP-H5N8 or H5N8 segments caused a significant reduction in the expression levels of IL-6, compared to the control infected group and the PR8 vaccinated group. This shows the lack of induction of an important inflammatory cytokine and emphasizes the safety profile of these candidate forms of vaccines.

After DC recognize the viral proteins, IFN-α and β are produced. Beside their role in controlling infection in infected cells, IFN-β induce the antiviral state in neighbor uninfected cells to minimize virus spread. Reduced IFN-β levels are associated with reduced survival in mice and increased viral replication in the lungs [20]. This is consistent with data of this study in NS vaccinated group, which showed a reduction in the level of IFN-β post challenge and high virus shedding in the lungs of infected chickens.

Despite that PA-vaccinated group induced IFN-β expression post infection, the influenza PA protein is known to antagonize the IFN-β [21]. The PA-X protein prevents the early accumulation of type 1 interferon response [22]. Only PR8_NP-H5N8_, PR8_NS-H5N8_, PR8_HA-H5N8_, and PR8_HA/NA-H5N8_ groups showed reduced levels of IFN-β post infection. The reduced levels of IFN-β in the PR8_NS-H5N8_ group could be related to the PDZ motif present on the C terminus in NS1 of H5N8 virus [23] which is responsible for suppression of IFN-β antiviral activity [24].

NS1 protein’s role is to interfere with the innate sensors by inhibiting almost all stages of antiviral pathways. The HPAI H5N8 virus used in this study harbors 103F and 106M in NS1, along with the presence of the PDZ motif at the C terminal, which inhibit the activation of the IFN-β promoter and suppress expression of other cellular genes by blocking posttranscriptional processing of cellular mRNAs [25]. This ensures the complete suppression of the antiviral host response. The NS of PR8 encodes for the PDZ motif, but not the 103F and 106M mutations, and thus it suppresses the IFN-β but not the other cellular pathways and mechanisms needed for antiviral activity [25].

IFN-β/α induce the antiviral interferon-stimulated genes (ISGs) which encode for proteins controlling multiple stages in viral replication by attenuating the expression of NP and M1 [26]. In this experiment, the level of IFNβ/α was downregulated in the challenged PR8_NS-H5N8_ vaccinated group. Therefore, NS of H5N8 is not a good candidate to be used in vaccines, especially the live attenuated forms [26].

The HPAI H5N1 virus’s PB1 segment expresses the PB1-F2 protein, which was shown to attenuate the pathogenicity of HPAI viruses when compared to HPAI viruses not expressing PB1-F2 in chickens [27]. The PB1-F2 of the parental HPAI H5N8 virus used in this study has 66N, not S. The 66S-infected mice had a delay in the early antiviral immune response up to 3 dpi by delaying the activation of type 1 interferons signaling genes and the expression of IFN-β [28]. In this experiment, the PR8_PB1-H5N8_ and the LP-H5N8 vaccinated groups showed upregulation in IFN-β and IFN-γ at 3 dpi.

The three interferons showed very high expression rates post infection in the groups vaccinated with PR8_PB1-H5N8_ and PR8_PA-H5N8_, despite the role of the H5N8 virus infection in shutting down the antiviral response of the host, which might control the infection and improve the survival rates. The elevation of IFN-β and IFN-α confers more antiviral protection against influenza virus infection but is not essential for the induction of apoptotic and certain inflammatory genes [29]. On the other hand, when treating the cells with IFN-α and γ prior to infection with the HPAI H5N1 virus, the virus titers in treated and non-treated cells were comparable [30,31]. Therefore, the protection might be more related to IFN-β than IFN-α or IFN-γ.

The reduction in the level of IFN-γ in PR8_NS-H5N8_ vaccinated chickens post challenge might explain the reduced survival rate due to reduced CTL motility and cytotoxicity [18].

The PR8_NS-H5N8_ and PR8_NP-H5N8_ vaccinated groups showed the lowest IFN-γ levels post challenge and the highest virus shedding in the lungs. These data show that the vaccine efficacy is indicated by the inverse correlation between the expression level of IFN-γ and the lung viral load [32,33].

PR8_NP-H5N8_ showed upregulation of (IL-2, IL-8 and IFN-α). All these factors could enhance the infection control in survived chickens. Previous work showed that infection with HP influenza viruses such as H5N1 and H5N6 is characterized by the elevation of IL-6 and IL-8, along with the interferons as contributing factors to apoptosis [34,35,36,37]. Infection with the LPAI viruses induced a milder response and downregulation [38] of these proinflammatory cytokines [39,40]. This elevation might be the key to higher pathogenicity [41]. IL-2 expressed in response to influenza infection increases lung inflammation and enhances natural killer cells [42]. Our data support that claim. PR8 is a low pathogenic virus that, despite killing all infected chickens, showed downregulation of IL-8 and interferon α and γ expression levels, accompanied by prolonged incubation before death and low cloacal shedding. This could also explain the low survival rate in the PR8_NA-H5N8_ group post challenge with HP H5N8, which showed the upregulation of IL-2 (significant), IL-8 (non-significant), and interferons.

IL-8 is elevated in response to infection by influenza viruses and induced by apoptosis [43]. IL-8 recruits neutrophils whose infiltration can cause acute inflammation, lung injury, and tissue damage [43,44,45]. On the other hand, induced neutrophils participate in antigen presentation to anti-viral effector CD8^+^ T cells [45]. IL-8 reduced expression in all vaccinated and challenged groups compared to the infected control group might explain the reduced mortality in groups vaccinated using H5N8 segments and the prolonged duration before death in the PR8 group.

In conclusion, our data indicate that including the HA segment remains essential for an efficacious homologous vaccine, and that influenza vaccination reduces IL-8 expression, while using single genes of a homologous parental challenge virus in vaccination also controls the expression of proinflammatory IL-6 and thus might enhance the protection capacity. More work needs to be carried out, and more cytokines need to be screened to determine the exact mechanism of protection and whether this finding is specific to H5N8 or applicable to other subtypes.

## 4. Materials and Methods

### 4.1. Viruses

Following two rounds of plaque purification, the eight gene segments of the HPAI H5N8 virus (A/green-winged teal/Egypt/871/2016), clade 2.3.4.4, were amplified by reverse transcription (RT) polymerase chain reaction (PCR), along with the segments of PR8 H1N1 virus (A/Puerto Rico/8/34), to be used to prepare the recombinant viruses used in this experiment. The H5N8 virus’s cleavage site multi-basic amino acid sequence (PLREKRRKR/GLF) was altered into a monobasic form (ETR/GLF) as previously described [46].

Each of the eight gene segments of each virus were then cloned into a reverse genetics plasmid-based system (pHW2000 vector, kindly provided by Richard Webby, St. Jude Children’s Research Hospital, Memphis, USA through Materials Transfer Agreement (MTA)) after being digested either with BsmBI for PB1, PA, HA, NP, M, and NS segments or BsaI for PB2 and NA segments, then transformed into DH5α competent cells (Thermo Fisher Scientific, Waltham, MA, USA) according to the manufacturers’ protocol. Plasmids were then confirmed by sequencing and digestion.

### 4.2. Cells and Reverse Genetics

Madin Darby canine kidney (MDCK) cells were cultured in Dulbecco’s Modified Eagle’s Medium (DMEM) (BioWhittaker, Lonza, Germany), while 293T human embryonic kidney cells were cultured in Opti-MEM medium (Gibco, Thermo Fisher Scientific) at 37 °C under 5% CO_2_. Both media were supplemented with 5% inactivated fetal bovine serum and 1% antibiotic antimycotic mixture (BioWhittaker, Lonza, Koln, Germany). Post confluency, a coculture of 293T and MDCK cells (3:1 ratio) was prepared in Opti-MEM free medium. After 24 h, constructs were then used to generate recombinant viruses as previously described [47,48]. The panel of generated reassorted viruses is listed in Figure 1.

The harvested viruses were inoculated in the allantoic cavities of 11-day-old specific pathogen-free embryonated chicken eggs (SPF-ECE) for propagation. Viruses were harvested at 48 h post infection (hpi) and then titrated by HA and stored at −80 °C. All forms were compatible and tested positive, except for (PB2 H5N8 + 7PR8). After two passages of propagation in SPF eggs, the gene constellation of each of reassortant viruses was confirmed by partial sequencing of each segment. Sequencing-confirmed viruses were then titrated using HA and stored at −80 °C.

### 4.3. Vaccine Preparation and Vaccination of Chickens

HA titers of the 10 viruses were individually adjusted using phosphate buffered saline to 6 log_2_ HA/50 µL. Viruses were inactivated by the addition of 0.1% formalin overnight, then mixed with Montanide ISA 71 VG (Seppic, Courbevoie, France) in the ratio recommended by the manufacturer (30 antigen/70 adjuvant *W*/*W*). A total of 220 specific-pathogen-free (SPF) Lohmann White chickens (4-week-old) were divided into 11 groups (20 chickens/group): 10 groups for reassorted and control viruses and one group for non-vaccinated chickens. Twenty random chickens were selected to collect serum samples to test for maternal immunity by HI assay. Chickens were then vaccinated by intramuscular injection with 0.5 mL of each inactivated vaccine containing equal HA units into the thigh. Ten random chickens were chosen to collect serum samples each wpv for four weeks.

A booster dose was administrated at 4 wpv, and serum samples were collected for two more wpv prior to challenge infection. At 2 wpv, three chickens of each group were dissected, and lungs were collected and stored at −80 °C.

### 4.4. Titration of HP H5N8 Virus in SPF-ECE and Challenge Infection

The purified HPAI H5N8 virus was used to infect SPF-ECE to determine the EID_50_/100 µL. The allantoic fluids were harvested and titrated using HA. The EID_50_ titer was calculated according to the Reed and Muench method.

The challenge infection was performed in 14 chickens of each vaccinated group at 6 wpv using natural routes (intraocular, intranasal, and intratracheal) by administrating a dose of 100 μL of 7.5 log_10_ EID_50_ of HPAI H5N8. Ten non-vaccinated chickens were infected while the remaining chickens were then monitored daily for 10 dpi. Cloacal and oral swabs were collected at 2 and 4 dpi to determine viral shedding. At 3 dpi, three chickens from each challenged (vaccinated and non-vaccinated) groups were dissected, and lungs were harvested.

### 4.5. HI and Neutralization Assay of Collected Serum

Collected serum samples (post vaccination and post challenge infection) were subjected to HI assay and two protocols of VMN assay against the parental HPAI H5N8 virus. In the first neutralization assay, log_2_ serially diluted sera were incubated with 200 TCID_50_ diluted virus for 1 h prior to infection of MDCK cells. Then, the virus serum mix was removed and 200 μL of infection media were added to the cells and incubated for three days. In the second assay, cells were first infected with 200 TCID_50_ virus for 1 h, then the virus inoculum was removed, and 200 μL of infection media containing the serially diluted serum was incubated with cells for two days. In both assays, inhibition was determined using HA assay.

### 4.6. RNA Extraction and qRT-PCR of Cytokines

Lungs were subjected to total RNA extraction using RNAeasy kit (Qiagen, Hilden, Germany) according to the manufacturer’s protocol. Total RNA concentration was measured using Nanodrop. Then, the RT of total RNA (200 ng) was performed using the Revert Aid First Strand cDNA Kit (Thermo Fisher Scientific) using random hexamers according to the manufacturers’ instructions and stored at −20 °C.

Quantitative real-time PCR (qRT-PCR) was performed using a Maxima SYBR Green qPCR Master Kit (2x) (Thermo Fisher Scientific). Sequences of selected cytokine primers are listed in Table 1. Melting curves were set post the end of the last PCR cycle. The analysis of relative expression was performed using β-Actin as the housekeeping control gene using the 2^(−ΔΔCT) equation.

ΔΔCT of vaccinated groups (at 6 wpv) = ΔCT (vaccination, challenge infection) − ΔCT (vaccination), where ΔCT (vaccination) = CT_V_ (target gene) − CT_V_ (B-Actin), and ΔCT (vaccination, challenge infection) = CT_VI_ (target gene) − CT_VI_ (B-Actin).

ΔΔCT of control infection groups = ΔCT (Control infection) − ΔCT (Control non-infected/non-vaccinated) [49,50]. Viral RNA shedding was also measured in the lungs by qRT-PCR to calculate RNA copy numbers.

### 4.7. Animal Experiments and Ethics Approval

Animal experiments were approved by the Ethics Committee of the National Research Centre (Protocol no. 18040). Experimental infection was performed under controlled laboratory and biosafety conditions at negative-pressure biosafety level 3 chicken isolators (Plas Labs, Lansing, MI, USA). Any chicken that showed a rapid onset of paralysis, disorientation, reluctance to feed, lethargy, or loss of body weight was culled as a humane endpoint.

### 4.8. Statistical Analysis

GraphPad Prism V5 (GraphPad Inc., San Diego, CA, USA) was used for statistical analysis. Statistical analysis was performed using the one-way ANOVA test, followed by Bonferroni post hoc testing. Data were represented as mean ± SD. *p* values of < 0.05 were considered statistically significant.

## Figures and Tables

**Table 1:** Sequences of primers used to detect cytokines expression as used in the RT-PCR.

Gene	Primer	Sequence (5′→3′)
IL-2	forward	TTG GCT GTA TTT CGG TAG CA
	reverse	GTG CAC TCC TGG GTC TCA GT
IL-6	forward	ATC CGG CAG ATG GTG ATA AA
	reverse	CCC TCA CGG TCT TCT CCA TA
IL-8	forward	CAT CAT GAA GCA TTC CAT CT
	reverse	CTT CCA AGG GAT CTT CAT TT
IFN-α	forward	GAC ATG GCT CCC ACA CTA CC
	reverse	AGG CGC TGT AAT CGT TGT CT
IFN-β	forward	GCT CAC CTC AGC ATC AAC AA
	reverse	GGG TGT TGA GAC GTT TGG AT
IFN-γ	forward	TGA GCC AGA TTG TTT CGA TG
	reverse	CTT GGC CAG GTC CAT GAT A
βActin	forward	CAC AGA TCA TGT TTG AGA CCT T
	reverse	CAT CAC AAT ACC AGT GGT ACG

## Data Availability

Not applicable.
